# Optimal allocations for two treatment comparisons within the proportional odds cumulative logits model

**DOI:** 10.1371/journal.pone.0250119

**Published:** 2021-04-21

**Authors:** Mirjam Moerbeek

**Affiliations:** Department of Methodology and Statistics, Utrecht University, Utrecht, The Netherlands; Roswell Park Cancer Institute, UNITED STATES

## Abstract

This paper studies optimal treatment allocations for two treatment comparisons when the outcome is ordinal and analyzed by a proportional odds cumulative logits model. The variance of the treatment effect estimator is used as optimality criterion. The optimal design is sought so that this variance is minimal for a given total sample size or a given budget, meaning that the power for the test on treatment effect is maximal, or it is sought so that a required power level is achieved at a minimal total sample size or budget. Results are presented for three, five and seven ordered response categories, three treatment effect sizes and a skewed, bell-shaped or polarized distribution of the response probabilities. The optimal proportion subjects in the intervention condition decreases with the number of response categories and the costs for the intervention relative to those for the control. The relation between the optimal proportion and effect size depends on the distribution of the response probabilities. The widely used balanced design is not always the most efficient; its efficiency as compared to the optimal design decreases with increasing cost ratio. The optimal design is highly robust to misspecification of the response probabilities and treatment effect size. The optimal design methodology is illustrated using two pharmaceutical examples. A Shiny app is available to find the optimal treatment allocation, to evaluate the efficiency of the balanced design and to study the relation between budget or sample size and power.

## Introduction

The randomized controlled trial is considered the gold standard for the comparison of multiple treatment conditions [1,2]. An important question while planning a trial is how many subjects should be allocated to each of the treatment groups. Most clinical trials use a balanced design so that treatment groups are of equal size. The main reason is that such a design often maximizes statistical power. Furthermore, it is consistent with the view of clinical equipoise that must exist before the start of the trial [3]. However, since long has it been known a balanced design is not necessarily the best choice, especially so when variances and/or costs vary across treatment conditions [4]. From a statistical point of view it is often more efficient to assign more subjects to the condition with lowest costs and highest variance.

Over the past two decades the literature on optimal treatment allocations has grown. For a quantitative outcome variable attention has been paid to two treatment comparisons [5–7] and comparisons of more than two treatments [3,8–11]. In addition, optimal allocations have been studied for cluster randomized trials [12–16] and other trials with clustered data [17–20].

Optimal treatment allocations have also been studied for binary outcome variables, including two treatment comparisons [21–23], comparisons of more than two treatments [24–26] and 2^*k*^ factorial designs [27]. Attention has also been paid to two treatment comparisons in cluster randomized trials [28], and trials with partially nested data [29] and to survival analysis models [30–32].

The derivation of optimal treatment allocations has gained less attention for trials with ordinal outcomes, while this type of outcome is omnipresent in the biomedical and social and behavioral sciences. Consider as an example a trial that compared different methods to treat schizophrenia [33]. The outcome was ordinal with four categories: normal, mildly or moderately ill, markedly ill, and severely ill. Another example is a trial that compared different drugs for the treatment of trauma. The outcome describes the clinical outcome: death, vegetable state, major disability, minor disability and good recovery [34]. Only two papers study optimal treatment allocations for ordinal outcomes [35,36]. Both of them focus on D-optimality, meaning the optimal treatment allocation minimizes the volume of the confidence ellipsoid of all parameter estimates. Ordinal data are often analyzed using an ordinal logistic model; the parameters of this model are the thresholds, treatment effect and eventually effects of predictive covariates. Thresholds are parameters that separate the ordinal categories; they are often not of primary interest. The treatment effect quantifies the difference between the treatments in term of their logits; this parameter is often of primary interest, so optimal designs that solely focus on the treatment effect are also of importance. An obvious optimality criterion is to minimize the variance of the treatment effect estimator, so that the optimal treatment allocation results in the most efficient design with respect to the treatment effect, and hence highest power for the test on treatment effect. This optimality criterion was indeed used in most of the previously cited references on optimal designs for quantitative or binary outcomes. The relation between sample size and power for a given treatment allocation has been studied previously [37], but attention has not been paid on the *optimal* treatment allocation.

The aim of this contribution is to study optimal treatment allocations for trials with ordinal outcomes. A restriction is made to two treatment conditions; both may be active treatments, or the one may be an active treatment and the other a placebo. This paper assumes proportional odds, meaning that the treatment effect size is constant across the cumulative logits of the ordinal logistic model. This assumption will be further explained in the next section. The focus is on the design that results in the most efficient estimate of the treatment effect. The optimal design is sought so that minimum variance, and hence maximum power, is achieved for a given total sample size or given budget. The effects of the number of categories, response probabilities across categories, treatment effect size and costs on the optimal allocation are explored. In addition, the efficiency of the balanced design as compared to the optimal design is discussed. Furthermore, the robustness of the optimal design against misspecification of the model parameters is evaluated. A Shiny App is made available for calculating the optimal treatment allocation for three to seven categories and for evaluating the efficiency of alternative allocations. This app also shows the effect of misspecification of the true value of the treatment effect and shows the relation between power and budget. The optimal design methodology is illustrated on the basis of two illustrative examples.

## Method

### Statistical model

The proportional odds ordinal logistic model is an extension of the logistic model for binary outcomes and is described in full detail elsewhere [38–40]. The outcome variable *Y*_*i*_ of subject *i* = 1,…,*N* is ordinal with categories *c*_1_,…,*c*_*J*_ and related probabilities *π*_*j*_ = *p*(*Y*_*i*_ = *j*) with $\sum_{j}^{J}\pi_{j}=1$. The *J*−1 non-redundant cumulative probabilities $\pi_{1}^{*},\ldots ,\pi_{j-1}^{*}$ are defined as $\pi_{j}^{*}=p(Y_{i}\leq j)$ and for each of these a logistic regression model is formulated

$$log\left(\frac{\pi_{j}^{*}}{1-\pi_{j}^{*}}\right)=\eta_{j}=\gamma_{j}+x_{i}^{\prime }\beta_{j}.$$ (1)

The thresholds *γ*_*j*_ are ordered such that −∞<*γ*_1_<⋯<*γ*_*J*−1_<∞. The predictor *x*_*i*_ is the treatment dummy with value 0 for the control and value 1 for the intervention and *β*_*j*_ is the vector of associated effects. The proportional odds assumption states that these effects are the same for all cumulative logits: *β*_*j*_ = *β*∀*j*. So instead of estimating *J*−1 treatment effects, one common treatment effect is estimated. Hence, the proportional odds model is a more parsimonious model than the non-proportional odds model, especially so when the number of categories is large. The proportional odds assumption can be tested by means of the Brant test [41].

The model parameters ***θ***′ = (*γ*_1_, *γ*_2_,…,*γ*_*J*−1_, *β*) can be estimated by means of maximum likelihood estimation, for further details see Glonek and McCullogh [42]. The associated covariance matrix $cov(\hat{\theta })$ plays an important role in the derivation of the optimal design. An asymptotic approximation is calculated from the inverse of the Fisher information matrix *F*, with

$$F=n_{C}F_{C}+n_{I}F_{I},$$ (2)

where *n*_*C*_ and *F*_*C*_ are the sample size and Fisher information matrix for the control and *n*_*I*_ and *F*_*I*_ are those for the intervention. The appendix explains how the matrices *F*_*C*_ and *F*_*I*_ are calculated as a function of the response probabilities.

### Derivation of the optimal treatment allocation

Optimal design methodology is used to derive the optimal allocation to treatments [43–45]. In this contribution the optimal allocation is sought under the condition

$$minimize\;var(\hat{\beta }).$$ (3)

To find the optimal allocation ratio, a constraint must be used, such as a budgetary constraint. The costs of enrolling, treating and measuring subjects may not exceed some pre-defined budget *B*. This constraint is formulated as *c*_*C*_*n*_*C*_+*c*_*I*_*n*_*I*_≤*B*, where *n*_*C*_ and *n*_*I*_ are the number of subjects in the control and intervention condition and *c*_*C*_ and *c*_*I*_ are the costs per subject in these two treatments. These costs include incentives, costs for recruitment, treatment and measurement, and may vary across the two treatments. Values of these costs should be available before the optimal design is derived. If there exists doubt on some of these values, then the most plausible value (based on similar studies in the past or the literature) may be used.

A special case of the constraint is when *c*_*C*_ = *c*_*I*_ = 1 and *B* is replaced by *N*. In that case the optimal design is sought under a fixed total sample size *N*: *n*_*C*_+*n*_*I*_ = *N*. This constraint is relevant when treatments for a rare disease are compared and only a limited number of subjects can be enrolled in the trial.

The optimal design p*∈(0,1) is the optimal proportion of subjects in the intervention condition. It is found numerically as follows: for any proportion *p* in the interval (0,1) the sample sizes *n*_*C*_ and *n*_*I*_ are calculated from the budgetary constraint. Note that these sample sizes are not necessarily integers. These sample sizes are plugged in the Fisher information matrix *F* from equation (2) and the value of the optimality criterion is calculated. The optimal design is the one with the smallest value on the optimality criterion. It can be shown not to depend on the budget *B* or total sample size *N*. In the remainder of this paper a step size of 0.01 for the proportion is used to find the optimal design. As the optimal design is expressed in terms of a proportion, the optimal design is a so-called continuous or approximate optimal design. For implementation, the optimal design has to be expressed in terms of the sample sizes in both conditions, a so-called discrete or exact optimal design. Exact designs are often difficult to find. In practice, the exact optimal design is approximated by calculating *n*_*I*_ = p*N and rounding to the nearest integer value and calculating *n*_*C*_ = *N*−*n*_*I*_.

Note that it is also possible to minimize the budget (or total sample size) so that a certain value for the variance of the treatment effect estimator, and hence a certain power for the test on treatment effect, is achieved. This will result in the same optimal proportion p* as when the variance is minimized given a fixed budget *B* (or total sample size *N*).

The performance of all other designs *p* as compared to the optimal design p* can be expressed in terms of their efficiencies. The efficiency of a design is calculated as

$$Eff=\frac{{var(\hat{\beta })}_{p=p^{*}}}{{var(\hat{\beta })}_{p=p}}.$$ (4)

The efficiency varies between 0 and 1; high values such as 0.8 or 0.9 are generally preferred.

A Shiny App is available to help the reader find the optimal allocation for a specific study at hand and to evaluate the efficiency of other designs, including the balanced design. It considers three to seven ordered response categories (https://utrecht-university.shinyapps.io/OD_Ordinal/). The Shiny App also shows the relation between the power for the test on treatment effect and the budget, for both the optimal design and the balanced design. This graph shows what power level can be obtained for a given budget, or how large the budget should be to achieve a certain desired power level. To derive the optimal design a prior estimate of the treatment effect size must be given. The robustness graph shows how well the optimal design performs if this prior estimate is different from the population value. The code for this Shiny App is available at https://github.com/MirjamMoerbeek/OD_Ordinal.

## Results

### Optimal treatment allocations

**Fig 1:**
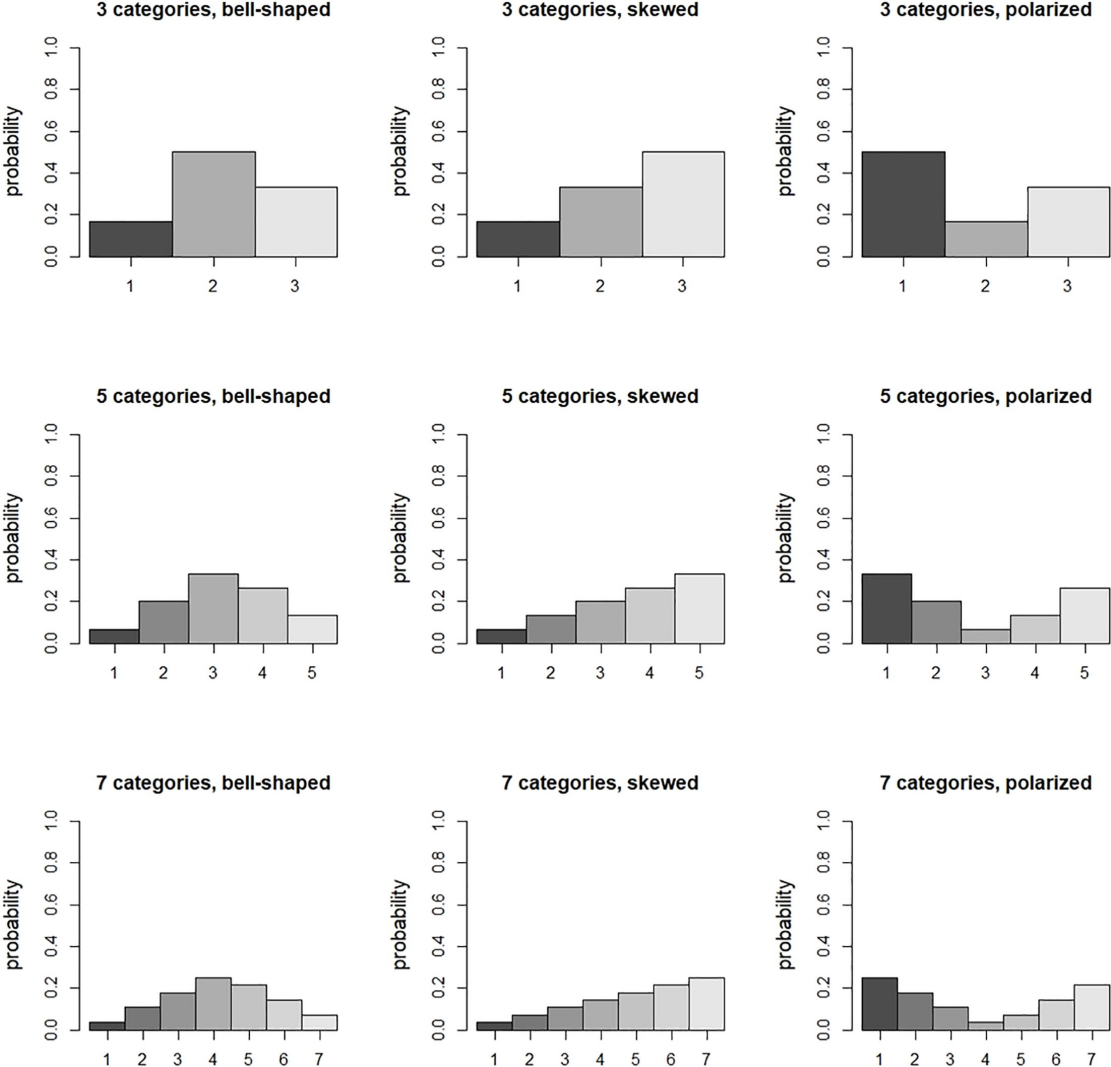
Response probabilities in the control condition.

This subsection shows how the number of response categories, the response probabilities in these categories, the treatment effect size and constraint influence the optimal allocation. Results are presented for three, five or seven categories. The response probabilities in the control condition are the same as those in Bauer and Sterba [46] and are depicted in Fig 1. Three distributions are taken into account: bell-shaped, skewed and polarized. The probabilities in the intervention follow from a proportional odds model with a treatment effect size (expressed as an odds ratio: *OR* = *e*^*β*^) of 1.68, 3.47 and 6.71, which are small, medium and large effect sizes [47]. A fixed total sample size is used (i.e. *c*_*I*_/*c*_*C*_ = 1), as well as a budgetary constraint with cost ratios *c*_*I*_/*c*_*C*_ = 2 and 5.

Tables 1–3 present the optimal proportion of subjects in the intervention and the efficiency of the balanced design for three, five and seven response categories, respectively. The following findings are observed with respect to the optimal proportion:

- For the polarized distribution the optimal proportion increases with increasing effect size, especially so for small number of categories. For the other two distributions the relation is either absent or slightly decreasing.
- The optimal proportion decreases when the cost ratio increases. This implies the optimal proportion for a fixed total sample size (i.e. *c*_*I*_/*c*_*C*_ = 1) is larger than for a fixed budget (i.e. *c*_*I*_/*c*_*C*_>1).
- For the polarized distribution the optimal proportion decreases with increasing number of categories. For the other two distributions the relation is absent
- The optimal proportion depends on the distribution of the response probabilities. It is highest for the polarized distribution.

**Table 1:** Optimal proportion of subjects in the intervention [efficiency of the balanced design] for three response categories.

cost ratio = 1^a^			
OR	bell-shaped	skewed	polarized
1.68	0.50 [1.00]	0.49 [1.00]	0.52 [1.00]
3.47	0.50 [1.00]	0.50 [1.00]	0.56 [0.99]
6.71	0.50 [1.00]	0.50 [1.00]	0.67 [0.96]
cost ratio = 2			
OR	bell-shaped	skewed	polarized
1.68	0.41 [0.97]	0.41 [0.97]	0.43 [0.98]
3.47	0.40 [0.97]	0.40 [0.97]	0.47 [1.00]
6.71	0.40 [0.96]	0.41 [0.97]	0.52 [1.00]
cost ratio = 5			
OR	bell-shaped	skewed	polarized
1.68	0.31 [0.87]	0.30 [0.86]	0.32 [0.89]
3.47	0.29 [0.86]	0.29 [0.86]	0.36 [0.94]
6.71	0.28 [0.85]	0.29 [0.86]	0.41 [0.98]

^a^Cost ratio = 1 implies a fixed total sample size.

**Table 2:** Optimal proportion of subjects in the intervention [efficiency of the balanced design] for five response categories.

cost ratio = 1^a^			
OR	bell-shaped	skewed	polarized
1.68	0.50 [1.00]	0.50 [1.00]	0.51 [1.00]
3.47	0.50 [1.00]	0.50 [1.00]	0.53 [1.00]
6.71	0.50 [1.00]	0.50 [1.00]	0.56 [0.99]
cost ratio = 2			
OR	bell-shaped	skewed	polarized
1.68	0.41 [0.97]	0.41 [0.97]	0.42 [0.97]
3.47	0.41 [0.97]	0.41 [0.97]	0.44 [0.99]
6.71	0.40 [0.97]	0.40 [0.97]	0.47 [1.00]
cost ratio = 5			
OR	bell-shaped	skewed	polarized
1.68	0.31 [0.87]	0.30 [0.87]	0.31 [0.88]
3.47	0.30 [0.86]	0.29 [0.86]	0.33 [0.90]
6.71	0.28 [0.85]	0.28 [0.85]	0.35 [0.93]

^a^Cost ratio = 1 implies a fixed total sample size.

**Table 3:** Optimal proportion of subjects in the intervention [efficiency of the balanced design] for seven response categories.

cost ratio = 1^a^			
OR	bell-shaped	skewed	polarized
1.68	0.50 [1.00]	0.50 [1.00]	0.50 [1.00]
3.47	0.50 [1.00]	0.50 [1.00]	0.52 [1.00]
6.71	0.50 [1.00]	0.50 [1.00]	0.54 [1.00]
cost ratio = 2			
OR	bell-shaped	skewed	polarized
1.68	0.41 [0.97]	0.41 [0.97]	0.42 [0.97]
3.47	0.41 [0.97]	0.41 [0.97]	0.42 [0.98]
6.71	0.40 [0.97]	0.40 [0.97]	0.44 [0.99]
cost ratio = 5			
OR	bell-shaped	skewed	polarized
1.68	0.31 [0.87]	0.31 [0.87]	0.31 [0.88]
3.47	0.29 [0.86]	0.29 [0.86]	0.31 [0.88]
6.71	0.28 [0.85]	0.28 [0.85]	0.33 [0.91]

^a^Cost ratio = 1 implies a fixed total sample size.

For a fixed total sample size the optimal proportion for the bell-shaped and skewed distribution is most often near 0.5 and the efficiency of the balanced design is higher than 0.95. For cost ratios *c*_*I*_/*c*_*C*_ = 2 and 5 the optimal proportion of subjects in the intervention is almost always less than 0.5. The further the optimal proportion is away from 0.5, the lower the efficiency of the balanced design. This efficiency is always larger than 0.8 for the chosen cost ratios. However, for larger cost ratios the efficiency further decreases. For instance, *c*_*I*_/*c*_*C*_ = 20 it is equal to 0.71 for a bell-shaped distribution with three categories and a small effect size. It can thus be concluded that the balanced design is not always the best choice.

### Robustness of the locally optimal designs

For any combination of the number of response categories and cost ratio, the optimal proportions in Tables 1–3 are locally optimal, meaning that they depend on the parameters of the ordinal logistic model: the thresholds (i.e. distribution of response probabilities) and treatment effect size [48]. For any cost ratio and number of categories, the optimal proportions for the bell-shaped and skewed distributions are rather similar and they show a weak or absent relation with the effect size. The optimal proportions for the polarized distribution are most often different from those of the other two distributions and are stronger related with the effect size.

To calculate the optimal design one must specify the response probabilities in each of the categories, along with the treatment effect size. Prior estimates can be obtained from an expert’s knowledge or expectations, the literature or a pilot study. However, there is always a risk these prior estimates are incorrect, meaning they are different from the population values. It is therefore important to assess the robustness of the locally optimal design against a misspecification of the prior estimates of the model parameters. For each combination of distribution of response probabilities and treatment effect size the efficiency of the optimal design was calculated for any other such combination. In other words, it was studied how well the optimal design for an incorrect prior estimate of these model parameters performs under the true population values of model parameters. Almost all optimal designs in Tables 1–3 have an efficiency of at least 0.9 for the other eight combinations of distribution of response probabilities and effect size. Only one of them has an efficiency that is lower than 0.9, but still above 0.85. It is the optimal design sought under a fixed total sample size, for the polarized distribution with three categories and OR = 6.71. The efficiency of this design is 0.88 or 0.89 in the case the distribution is bell-shaped or skewed with OR = 1.68 or OR = 3.47. In general it can be concluded that the locally optimal designs are highly robust with respect to misspecification of the model parameters.

For any study, the robustness graph in the Shiny app can be used to evaluate the robustness of an optimal design based on a prior estimate of the effect size if the population value is different from this prior estimate.

## Illustrative examples

### Example 1: Treatment for schizophrenia

**Fig 2:**
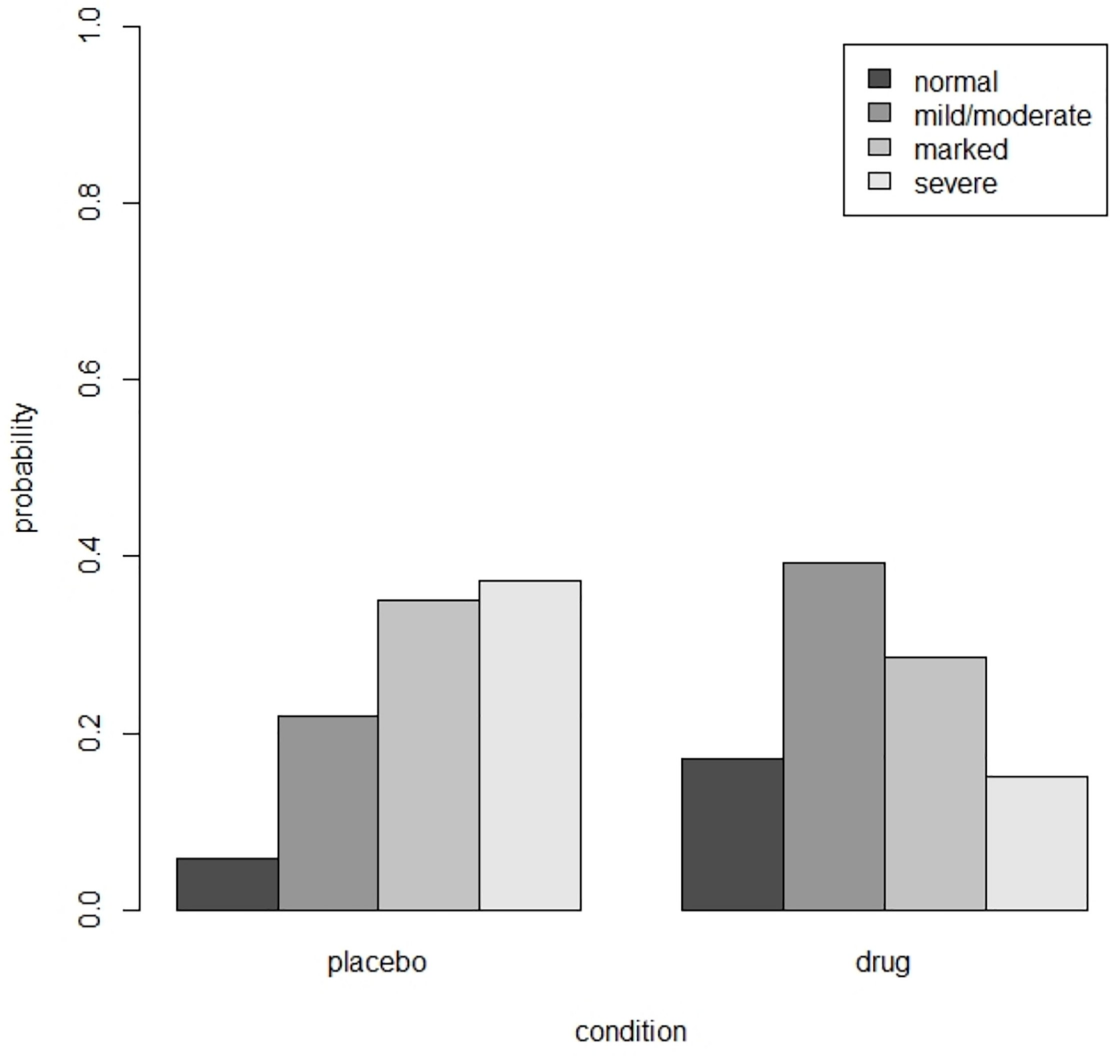
Estimated probabilities in the placebo and drug condition for the schizophrenia example.

The psychiatric example in section 10.3 of Hedeker and Gibbons [33] uses data from the National Institute of Mental Health Schizophrenia Collaborative Study. In this trial 108 schizophrenic patients were randomized to placebo and 329 to anti-psychotic drug. The drug group consisted of three different drugs, but since previous analyses revealed similar effects these drug groups were combined. The outcome ‘severity of illness’ was categorized into four ordered categories: normal, mildly or moderately ill, markedly ill, and severely ill. At baseline there were no differences between the two treatment groups with respect to the outcome ($\hat{\beta }=-0.09,\;p=0.406,\;OR=0.92$). Three weeks after baseline the outcome was observed on 87 patients in the placebo condition and 287 in the drug condition. A proportional odds model showed the effect of condition was significant ($\hat{\beta }=1.217,\;p<0.001,\;OR=3.377$). The estimated thresholds were ${(\hat{\gamma }}_{1},\;{\hat{\gamma }}_{2},\;{\hat{\gamma }}_{3})=(-2.79,-0.96,0.52)$, which corresponds to probabilities in the control (*p*_1_, *p*_2_, *p*_3_, *p*_4_) = (0.06,0.22,0.35,0.37). The estimated probabilities in both conditions are shown in Fig 2; better results are observed in the drug group. The Brant test shows the proportional odds assumption is not violated (*χ*^2^ = 3.13, *df* = 2, *p* = 0.21).

**Fig 3:**
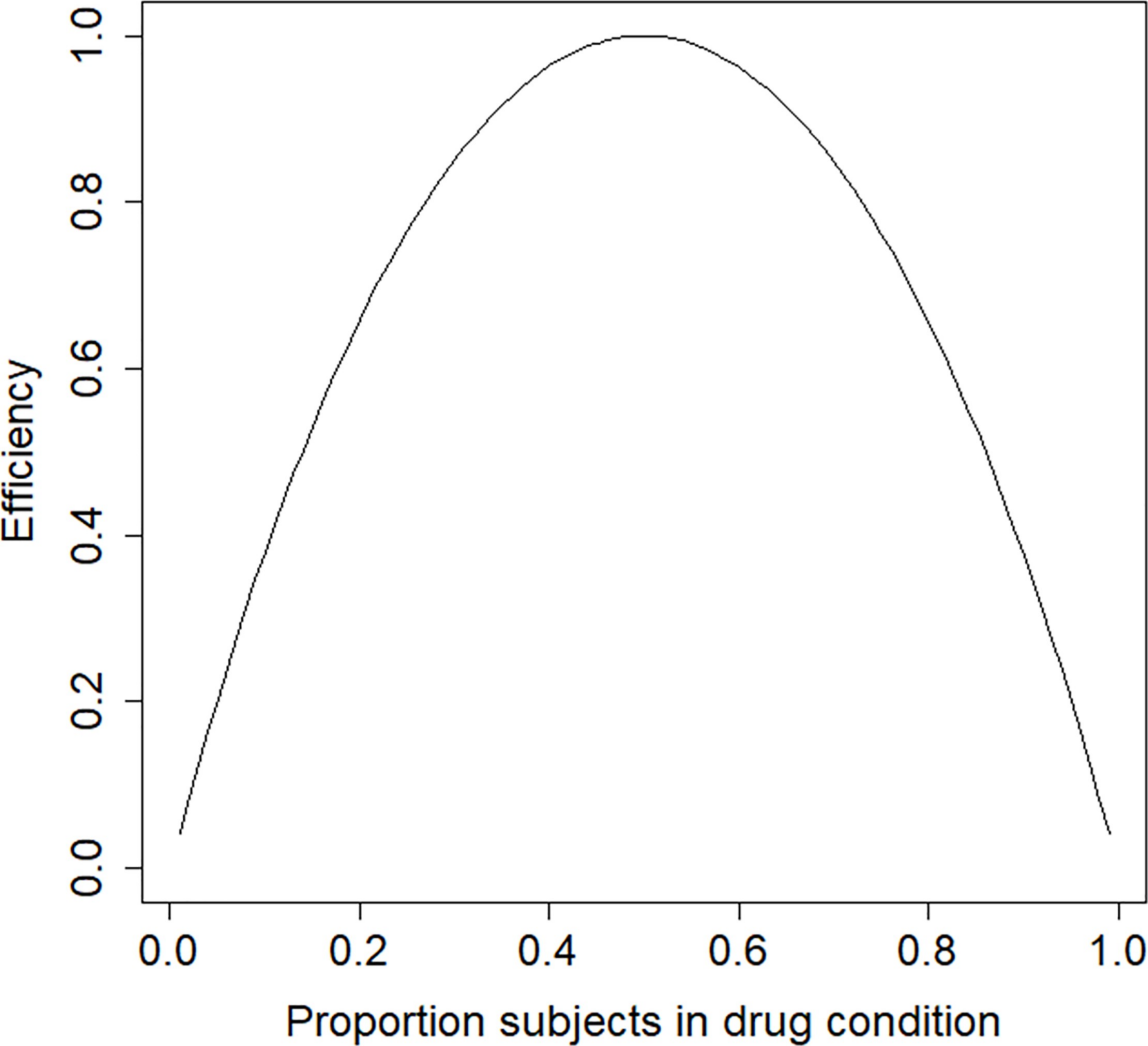
Efficiency graph for the schizophrenia example.

Suppose this trial is to be replicated such that the power for any given sample size is maximized. A relevant question is whether a design with an equal number of subjects per treatment condition is the most efficient. The design is locally optimal and the parameter estimates as given above are used to derive the optimal design. Fig 3 shows the efficiencies as a function of the proportion of patients in the drug condition. The optimal proportion is p* = 0.50, which implies the optimal design is the balanced design. This optimal design is highly robustness to an incorrect prior guess of the treatment effect: for any population value of the treatment effect in the range *OR* = [0.1,10] its efficiency is at least 0.96.

**Fig 4:**
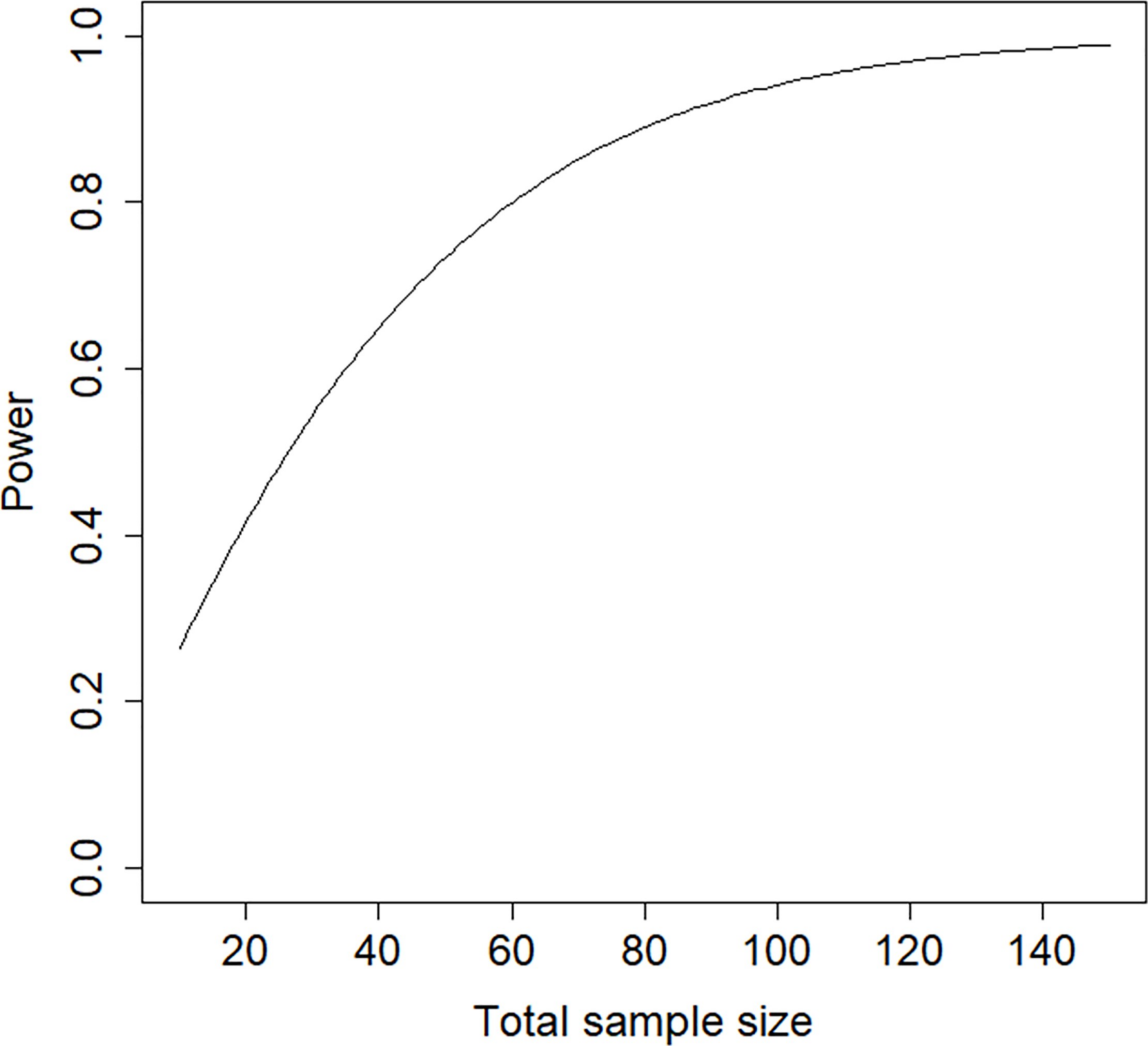
Power graph for the schizophrenia example.

A relevant question is how large the sample size should be to detect at treatment effect of size *β* = 1.217 with a power level of 0.8 in a test with type I error rate of 0.05 and a one-sided alternative hypothesis. Fig 4 shows the relation between total sample size and power. The total sample size can be calculated to be 61, which is much lower than in the original study.

### Example 2: Treatment for trauma

This example uses data from Chuang-Stein and Agresti [34]. Five ordered categories describe the clinical outcome of patients who experienced trauma: death, vegetable state, major disability, minor disability and good recovery. These are often called the Glasgow Outcome Scale. There were four treatment groups based on the dose level of the investigational medication: placebo (*n* = 210), low dose (*n* = 190), medium dose (*n* = 207) and high dose (*n* = 195).

**Fig 5:**
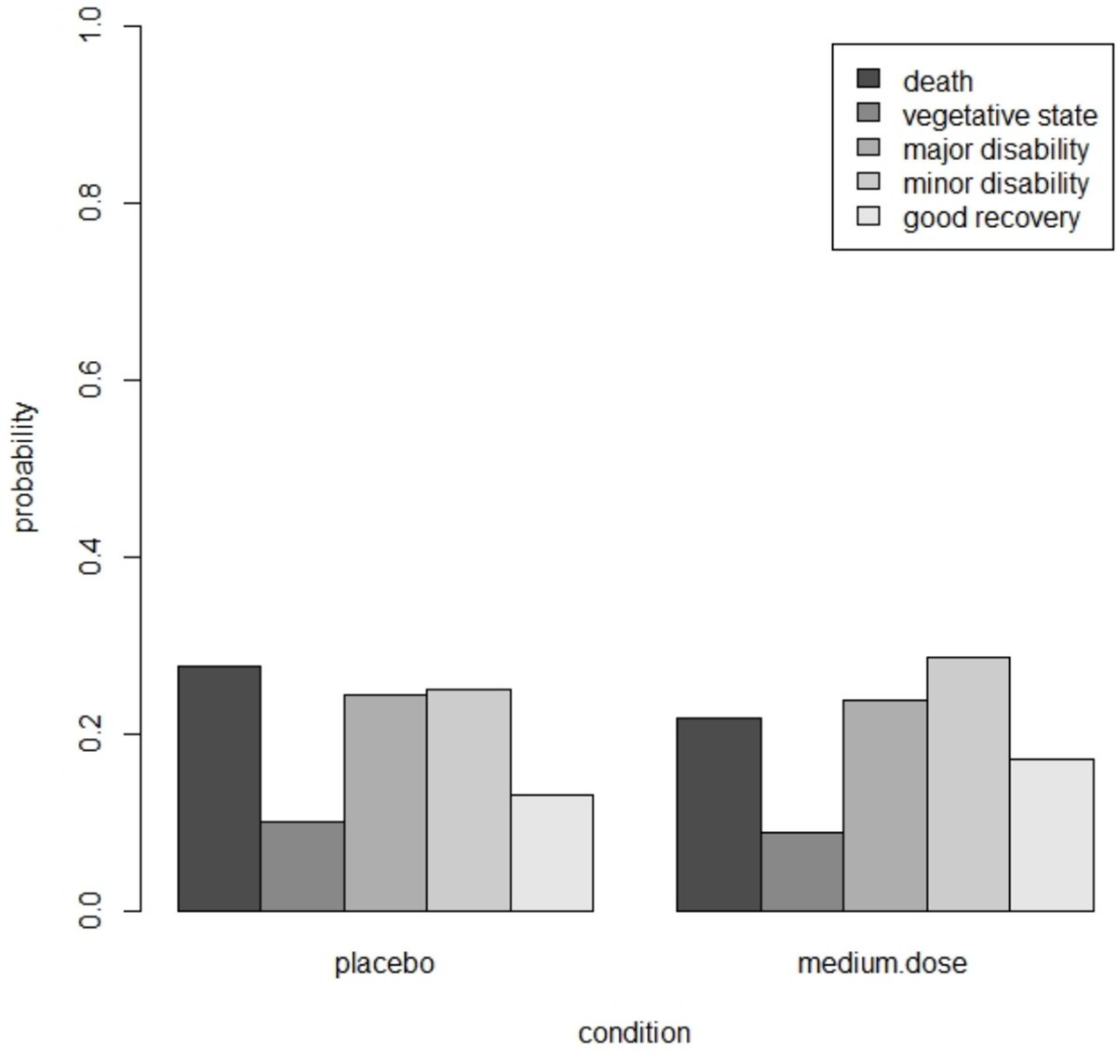
Estimated probabilities in the placebi and medium dose group in the trauma example.

Suppose this trial has to be replicated and interest is in the comparison of placebo versus medium dose. A proportional odds model shows the estimated treatment effect is of small size and insignificant at $\alpha =0.05\;(\hat{\beta }=-0.324,\;p=0.064,\;OR=0.723)$. The estimated thresholds are ${(\hat{\gamma }}_{1},\;{\hat{\gamma }}_{2},\;{\hat{\gamma }}_{3},\;{\hat{\gamma }}_{4})=(-0.95,-0.50,0.49,1.90)$, which corresponds to probabilities in the control (*p*_1_, *p*_2_, *p*_3_, *p*_4_, *p*_5_) = (0.28,0.10,0.24,0.25,0.13). The estimated probabilities in both conditions are shown in Fig 5; more favorable results are observed in the drug group. The proportional odds assumption is not violated (Brant test: *χ*^2^ = 4.86, *df* = 3, *p* = 0.18).

**Fig 6:**
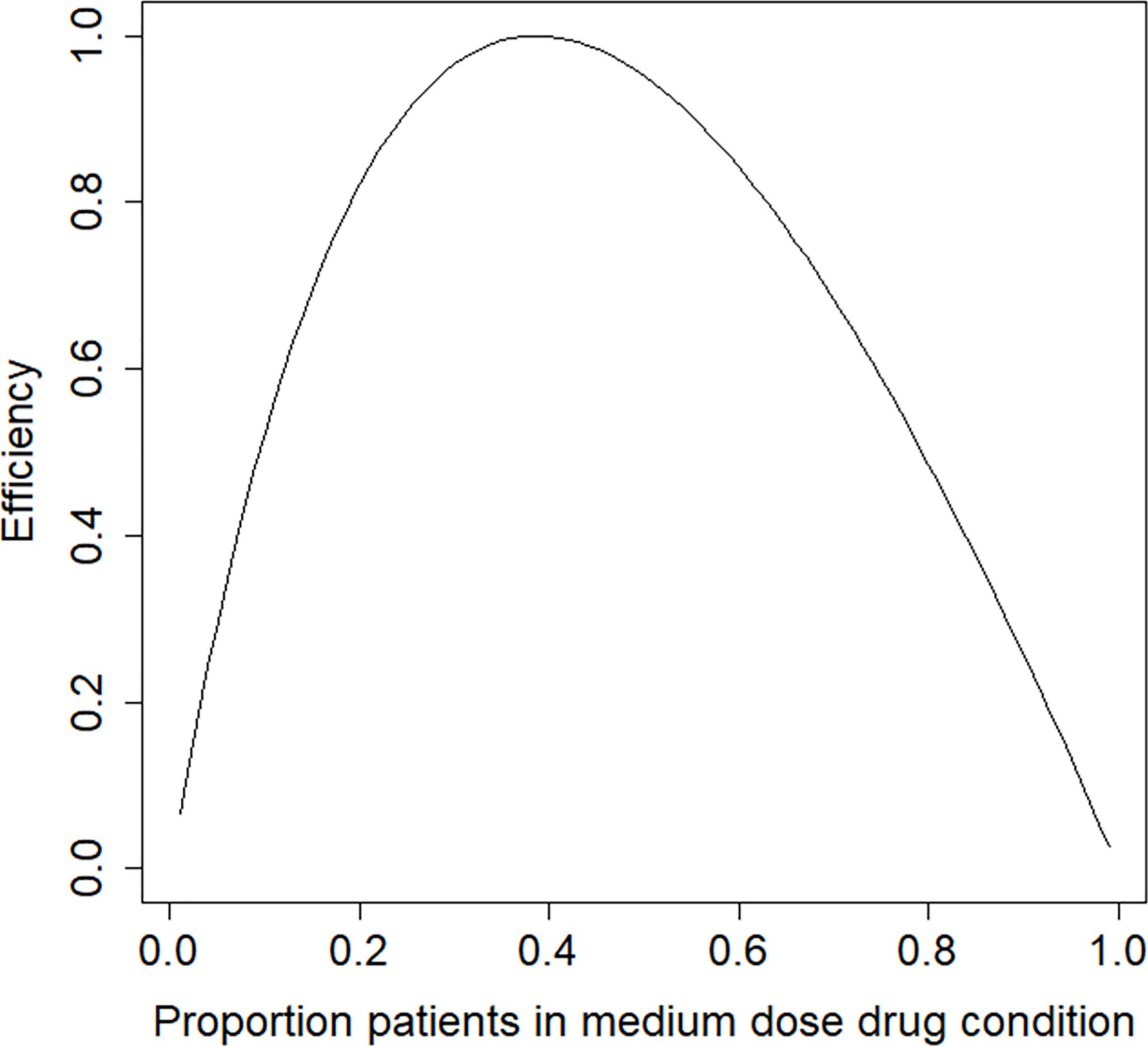
Efficiency graph for the trauma example.

In the derivation of the optimal design we assume differential costs across the two treatments, with a cost ratio *c*_*I*_/*c*_*C*_ = 2.5. The estimated treatment effect and thresholds are used to derive the locally optimal design. Fig 6 shows the efficiency plot. The optimal proportion of subjects in the medium dose drug condition is p* = 0.39 and the efficiency of the balanced design is 0.95. The balanced design is highly efficient but not the best choice. The optimal design is highly robust to an incorrect prior estimate of the treatment effect size. For any population value of the treatment effect in the range *OR* = [0.1,10] it has an efficiency of at least 0.99.

**Fig 7:**
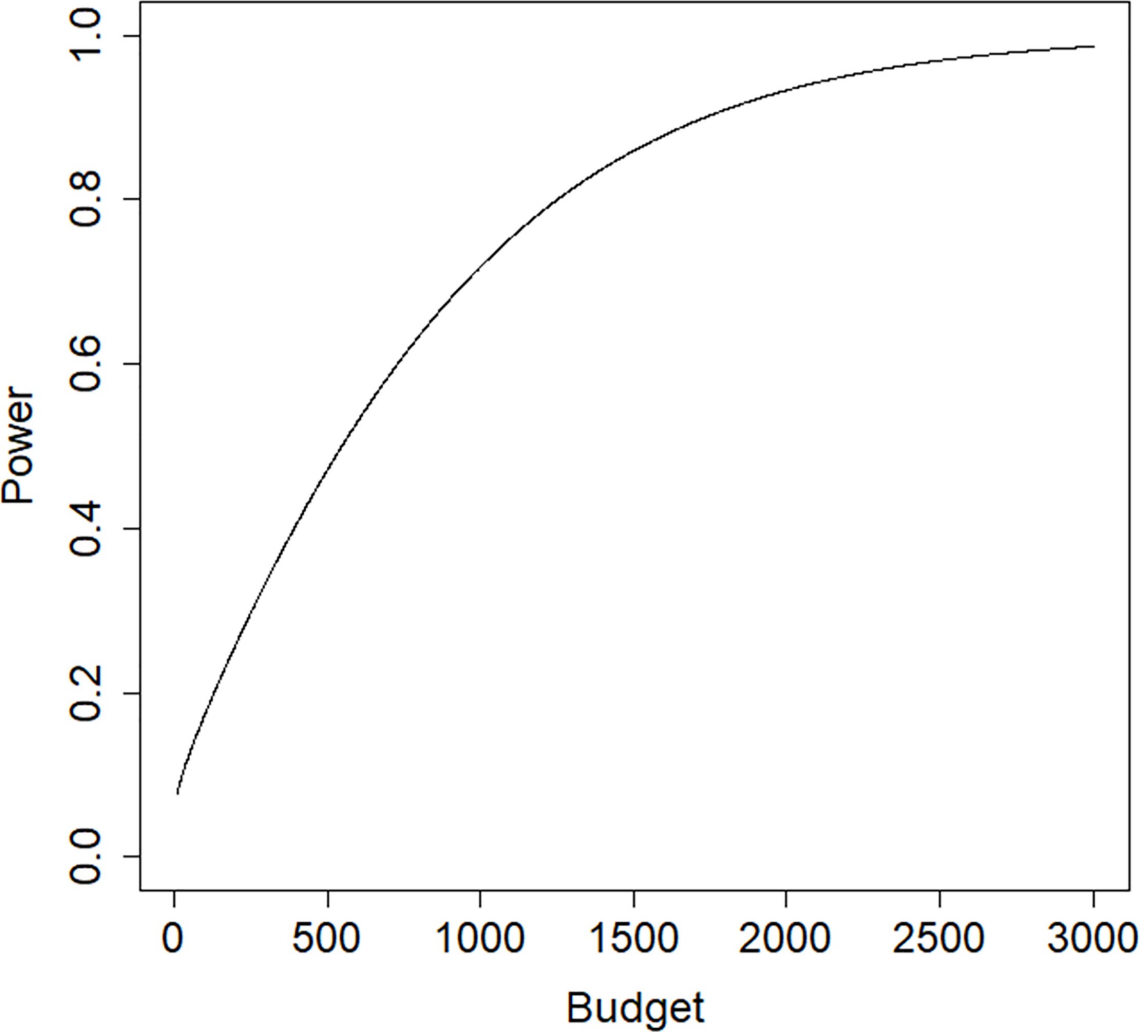
Power graph for the trauma example.

Fig 7 shows the relation between budget and the power to detect a treatment effect of *β* = −0.324 in a one-sided test with a type I error rate of 0.05. The costs are scaled so that *c*_1_ = 1; in other words, the budget is expressed in terms of the costs per subject in the control condition. To achieve a power of 0.8 for the test on treatment effect at a type I error rate of 0.05 and a one sided alternative a budget of *B* = 1262 is needed. The corresponding total sample size is 796, which is almost twice as high as in the original study.

## Conclusions and discussion

This study provides methodology and a Shiny app to calculate the optimal treatment allocation in a trial with two treatment conditions and ordinal outcomes. The optimal design minimizes the variance of the treatment effect estimator, so that the power for the test on treatment effect is maximized. The optimal design is sought under a budgetary constraint, of which a fixed total sample size is a special case.

In general the optimal proportion subjects in the intervention condition decreases with the cost ratio. For the polarized distribution the optimal proportion increases with increasing effect size and decreases with increasing number of response categories, while for the other two distributions these relations are decreasing or absent. Furthermore, the optimal proportion depends on the distribution of response probabilities: it is highest for the polarized distribution. However, the optimal design is highly robust with respect to misspecification of the parameters of the ordinal logistic model, hence it is not considered necessary to implement robust optimal designs, such as a maximin optimal design. The widely used balanced design has a high efficiency in the case the total sample size is fixed. However, its efficiency declines when the cost ratio increases. It can therefore be concluded the balanced design is not necessarily the best choice. These findings are also illustrated by the two examples. In the first a fixed total sample size was used as a constraint. The balanced design turned out to be the optimal design. In the second a budgetary constraint was used, resulting in a loss of efficiency of the balanced design.

The methodology in this paper can also be used if two naturally existing groups are to be compared on some ordinal outcome, such as males and females or smokers and non-smokers. In some studies the number of subjects in one of the groups may be small, for instance the number of subjects suffering from a rare disease. It may occur fewer of such subjects can be enrolled in the trial than dictated by the optimal design. In that case the thus unused part of the budget may be spend on sampling more subjects in the other group. The Shiny app may be used to evaluate the efficiency of such a sub-optimal design.

The optimal designs as presented in this paper are based on a model that uses cumulative logits: the probability of a response category and all previous categories is compared to the probability of all subsequent response categories. There exist other types of logit models for ordinal response variables, see section 3.1 and chapter 4 in Agresti [39]. The adjacent-categories logits are the log odds for pairs of adjacent variables: the probability of a response category is compared to the probability of the subsequent response category. The choice between cumulative and adjacent categories is based on whether one wants to refer to individual response categories or groupings of response categories that use the entire scale of response categories. The advantage of the cumulative logit model is that its effect estimates are approximately invariant to the choice and number of response categories. The continuation-ratio logits are calculated from the probability of a response category against either all higher categories or all lower categories. Such a model is useful when the ordinal response outcome is determined by a sequential process, such as different cancer stages or developmental stages. A special application is discrete-time survival analysis, where the time variable is discretized into a number of coarse intervals. The hazard probability of experiencing some event in a particular time interval is conditional on not having experienced the event in any previous intervals. Optimal designs for discrete-time survival analysis have been presented previously [32].

Future work may focus on the derivation of optimal treatment allocations for extensions to model (1). One such extension are factorial designs, for instance for evaluating pharmaceutical and behavioral interventions simultaneously. Another extension may focus on one treatment factor with more than two levels, for instance a placebo versus different types of drugs. Attention should also be paid to the non-proportional odds model, where the effect of treatment may vary across the cumulative logits. This implies *J*−1, rather than one single, treatment effects should be estimated, and this should be reflected in the optimality criteria. It may be then relevant to focus on a multiple-objective optimal design, where optimality criteria are ordered with respect to their relative importance. For instance, estimating the effect of treatment on the first cumulative logit, that separates the category “death” from all other categories, may be considered more important than the treatment effects on all other cumulative logits. It may also be of interest to study the robustness of the optimal designs as derived under the proportional-odds assumption against violation of this assumption. Other extensions include trials with nested data, such as cluster randomized trials and trials with partially nested data. An example of the latter is a trial with nesting of subjects within therapists in the psychotherapy condition and no nesting in the wait list control. Finally, the effect of predictive covariates on the optimal design should be studied.

For all these extensions it may turn out the locally optimal design is not robust against misspecification of the model parameters. In such cases one may derive a robust optimal design, such as a maximin design [23]. The optimal design is calculated for each combination of plausible values of the model parameters. Next, the efficiency of each other design as compared to the optimal design is computed. Finally, for each design the minimal efficiency is selected and the maximin optimal design is the one with the highest minimal efficiency. Instead of using a range of plausible values of model parameters, one may also specify a prior distribution to calculate a Bayesian optimal design [49]. An alternative is the use of an internal pilot [50]. The optimal design is first calculated based on a priori estimates of the model parameters. A proportion of the budget or total sample size is then used and pilot data are collected. The model is then fitted to the data and the estimates of the model parameters are used to re-calculate the optimal design. Finally all data, including those from the internal pilot, are analyzed and the effect of treatment is estimated. It may be clear this approach is especially suitable for trials with short duration.

In summary, this paper has provided optimal allocations for two treatment comparisons when the response variable is ordinal and modelled using a proportional odds cumulative logits model. The Shiny app enables researchers to find the optimal allocation for their trial, and to evaluate the efficiency of alternative designs, including the balanced design.

## Supporting information

S1 Appendix Calculation of Fisher information matrix. (DOCX)
