# Supplementary material for: Optimal allocations for two treatment comparisons within the proportional odds cumulative logits model
Source: PLoS One. 2021 Apr 21;16(4):e0250119. doi: 10.1371/journal.pone.0250119 (PMC8059828; doi:10.1371/journal.pone.0250119)
Supplement: S1 Appendix — (DOCX) [file pone.0250119.s001.docx]

# Appendix. Calculation of Fisher information matrix

This appendix is based on ^33^ and its supplementary materials.

The Fisher information matrix is the sum of the two Fisher information matrices in the two treatment conditions, weighted by the sample sizes

$$F=n_{C}F_{C}+n_{I}F_{I}.$$

The matrix $\boldsymbol{F}_{C}$ for the control condition follows from

$$\boldsymbol{F}_{C}=\left( \frac{\partial\boldsymbol{\pi}_{C}}{\partial\boldsymbol{\theta}^{T}} \right)^{T}diag{(\boldsymbol{\pi}_{C})}^{-1}\frac{\partial\boldsymbol{\pi}_{C}}{\partial\boldsymbol{\theta}^{T}}$$

with

$$\frac{\partial\boldsymbol{\pi}_{C}}{\partial\boldsymbol{\theta}^{T}}=\left( \boldsymbol{C}^{T}\boldsymbol{D}_{C}^{-1}\boldsymbol{L} \right)^{-1}\boldsymbol{X}_{C}.$$

The matrix $\boldsymbol{C}^{T}$ is a $J\times(2J-1)$ constant matrix given by

$$\boldsymbol{C}^{T}=\left( \begin{matrix} \boldsymbol{I}_{J-1} & \boldsymbol{-I}_{J-1} & 0_{J-1} \\ 0_{J-1}^{T} & 0_{J-1}^{T} & 1 \end{matrix} \right),$$

with $\boldsymbol{I}_{k}$ the identity matrix of order $k$ and $0_{k}$ a vector of $k$ zeros. The matrix $\boldsymbol{L}$ is a $(2J-1)\times J$ constant matrix given by

$$\boldsymbol{L}=\left( \begin{matrix} 1 & 0 & \ldots& 0 & 0 \\ 1 & 1 & 0 & \ldots& 0 \\ \vdots& \vdots& \ddots& \vdots& \vdots\\ 1 & 1 & \ldots& 1 & 0 \\ 0 & 1 & \ldots& 1 & 1 \\ 0 & 0 & 1 & \ldots& 1 \\ \vdots& \vdots& \ddots& \ddots& \vdots\\ 0 & 0 & \ldots& 0 & 1 \\ 1 & 1 & \ldots& 1 & 1 \end{matrix} \right).$$

Furthermore, $\boldsymbol{D}_{C}=diag(\boldsymbol{L}\pi_{C})$ is a $(2J-1)\times(2J-1)$ diagonal matrix.

$\left( \boldsymbol{C}^{T}\boldsymbol{D}_{i}^{-1}\boldsymbol{L} \right)^{-1}$ is the $J\times J$ matrix

$$\left( \boldsymbol{C}^{T}\boldsymbol{D}_{i}^{-1}\boldsymbol{L} \right)^{-1}=\left( \begin{matrix} \pi_{C1}(1-\pi_{C1}) & 0 & \ldots& 0 & \pi_{C1} \\ {-\pi}_{C1}(1-\pi_{C1}) & \pi_{C2}(1-\pi_{C2}) & \ddots& \vdots& \pi_{C1} \\ 0 & {-\pi}_{C2}(1-\pi_{C2}) & \ddots& 0 & \vdots\\ \vdots& \ddots& \ddots& \pi_{C,J-1}(1-\pi_{C,J-1}) & \pi_{C,J-1} \\ 1 & 1 & 1 & 1 & \pi_{CJ} \end{matrix} \right),$$

where$\pi_{Cj}$ is the probability for category *j* in the control condition. $\boldsymbol{X}_{C}$ is the $J\times J$ design matrix which is defined as

$$\boldsymbol{X}_{C}=\left( \begin{matrix} \boldsymbol{I}_{J-1\times J-1} & \boldsymbol{0}_{J-1\times J-1} \\ \boldsymbol{0}_{J-1}^{T} & \boldsymbol{0}_{J-1}^{T} \end{matrix} \right).$$

The matrix $\boldsymbol{F}_{I}$ for the intervention condition is calculated in a similar way. The probability $\pi_{Cj}$ in $\left( \boldsymbol{C}^{T}\boldsymbol{D}_{i}^{-1}\boldsymbol{L} \right)^{-1}$ should be replaced by the probability $\pi_{Ij}$ for the intervention condition and the design matrix is

$$\boldsymbol{X}_{I}=\left( \begin{matrix} \boldsymbol{I}_{J-1\times J-1} & \boldsymbol{I}_{J-1\times J-1} \\ \boldsymbol{0}_{J-1}^{T} & \boldsymbol{0}_{J-1}^{T} \end{matrix} \right).$$
